# Development and evaluation of an artificial intelligence-based electrocardiogram prediction model for emergency chest pain patients

**DOI:** 10.3389/fmed.2026.1746364

**Published:** 2026-03-10

**Authors:** Yazhi Luo, Juan Peng, Wen Peng, Qianqian Zhao, Guiling Li, Wanhong Liu

**Affiliations:** 1 Hubei Province Key Laboratory of Allergy and Immunology, Taikang Medical School (School of Basic Medical Sciences), Wuhan University, Wuhan, China; 2 The Second Xiangya Hospital of Central South University, Changsha, China

**Keywords:** artificial intelligence, chest pain, deep learning, electrocardiogram, emergency

## Abstract

**Background/Objectives:**

Rapid triage and etiological differentiation are critical for patients with acute chest pain in the emergency department. The 12-lead electrocardiogram (ECG), as a non-invasive, readily available, and cost-effective diagnostic modality, provides immediate information and serves as the first-line tool for clinical evaluation. However, ECG interpretation remains highly dependent on clinician expertise and is subject to inter-observer variability. Artificial intelligence (AI)-based analytical methods can deliver automated, consistent, and real-time assessment, thereby potentially enhancing diagnostic accuracy and facilitating timely clinical decision-making.

**Methods:**

This study included 1,188 patients with acute chest pain who visited the emergency department in the Second Xiangya Hospital of Central South University, between March 2024 and March 2025. Standard 12-lead ECGs, clinical information, and final diagnoses were collected. After data preprocessing, a convolutional neural network (CNN) incorporating a channel attention mechanism was developed and trained. Model performance was assessed using accuracy, precision, recall, F1-score, area under the curve (AUC), and confusion matrices. Additionally, a blinded comparative evaluation was conducted against expert cardiologists.

**Results:**

The model demonstrated excellent discriminative capability for ST-elevation myocardial infarction (STEMI) and non-ST-elevation myocardial infarction (NSTEMI), with AUC values of 0.986 and 0.916, respectively. For STEMI, all performance metrics indicated superior diagnostic accuracy, and inference time was significantly shorter than manual interpretation (0.24 ± 0.08 s, *p* < 0.001). However, detection performance for unstable angina (UA) and aortic dissection (AD) remained suboptimal, characterized by high sensitivity but relatively low precision.

**Conclusions:**

The deep learning model based on 12-lead ECGs enables rapid and reliable detection of STEMI and NSTEMI, highlighting its potential as a valuable clinical decision-support tool in emergency department. Nevertheless, the recognition of UA and AD remains limited due to non-specific or transient electrophysiological features.

## 1 Introduction

Acute chest pain is one of the most common complaints in the emergency department (ED), with etiologies ranging from benign to life-threatening conditions. Epidemiological data indicate that chest pain accounts for approximately 20% of all ED visits, posing substantial diagnostic and management challenges for clinicians (1). Conventional diagnostic workflows commonly include electrocardiography (ECG), serial assessments of cardiac biomarkers, and advanced imaging modalities such as computed tomography angiography (CTA) (2, 3), which may prolong diagnostic timelines. For patients with high-risk causes of chest pain, delayed intervention can result in increased mortality or severe sequelae.

In recent years, the rapid development of artificial intelligence (AI)-particularly in deep learning, computer vision, and natural language processing-has transformed medical data analysis and clinical decision support (4, 5). Numerous studies have confirmed the effectiveness of deep learning and machine learning algorithms in automated ECG interpretation (6–8). For instance, the Mayo Clinic research group (9) developed an AI-based ECG analysis system trained on more than 2.4 million 12-lead ECGs, achieving diagnostic performance comparable to experienced cardiologists in identifying arrhythmias, conduction abnormalities, and ischemic changes. Other investigations (10–12) have further demonstrated that AI-enabled ECG systems can markedly shorten diagnostic time, facilitate rapid risk stratification, and support timely and personalized clinical management.

However, despite these advances, AI models for acute chest pain remain underdeveloped. Existing algorithms often focus on single disease recognition, which does not fully meet the demands of complex, multi-etiological presentations in real world clinical practice. To address this gap, this study collected a large dataset of ECGs from chest pain patients in the ED to construct a clinically representative database. A deep learning model based on convolutional neural networks (CNN) was then developed using the PyTorch framework. The network incorporated multiple convolutional, pooling, batch normalization, and fully connected layers, enhanced with a channel attention mechanism to improve sensitivity to clinically relevant ECG features. This study aimed to evaluate the model's performance in differentiating major causes of acute chest pain, including ST-elevation myocardial infarction (STEMI), non-ST-elevation myocardial infarction (NSTEMI), unstable angina (UA), and aortic dissection (AD). By comparing the AI model's predictions with cardiologist interpretations, we aimed to validate its clinical feasibility and assess its potential to expedite triage and improve diagnostic efficiency in emergency care.

## 2 Materials and methods

### 2.1 Study design and clinical data

This study was conducted in the Emergency Department of the Second Xiangya Hospital of Central South University, between March 2024 and March 2025. A total of 1,188 patients presenting with acute chest pain who underwent standard 12-lead ECG examinations were enrolled. Comprehensive clinical data were collected, including demographic characteristics, ECG recordings, auxiliary laboratory and imaging results, and final confirmed diagnoses. Diagnostic labels included five categories: ST-elevation myocardial infarction (STEMI), non-ST-elevation myocardial infarction (NSTEMI), unstable angina (UA), aortic dissection (AD), or normal ECG. Final diagnostic labels were extracted from the hospital electronic medical record as the final confirmed diagnosis after completion of the routine emergency and inpatient workup. In this study, the task was formulated as disease-specific binary classification for emergency triage, where each model detects one target disease against normal ECGs. For UA and AD, ECG alone is not sufficient for definitive clinical diagnosis; these experiments are presented as exploratory analyses to assess the extent to which an ECG-report–only AI approach can capture disease-associated patterns under real-world data availability constraints, rather than to replace biomarker- or imaging-based diagnostic pathways.

Inclusion criteria were: (1) age ≥18 years; (2) presence of acute chest pain; and (3) admission to the emergency department.

Exclusion criteria were: (1) history of cardiac surgery within 3 months (e.g., valve replacement or pacemaker implantation); (2) current use of digoxin or antiarrhythmic agents; and (3) pregnancy or lactation.

All ECGs were recorded using a MAC 800 resting electrocardiograph with a sampling rate of 150 Hz, paper speed of 25 mm/s, voltage setting of 1 mV/10 mm, and a recording duration of 10 s.

### 2.2 Data preprocessing

All ECG data were fully anonymized prior to analysis to ensure the removal of personally identifiable information. Records containing missing values or significant waveform distortions were excluded. Additionally, signals exhibiting extreme heart rates (< 30 beats/min or >200 beats/min), as determined by QRS detection, were discarded to maintain physiological validity.

Raw ECGs were converted from PDF format to high-resolution PNG images (14,034 × 9,917 pixels), preserving clinically relevant waveforms while removing redundant background. Contour detection and clustering were applied to determine the minimum bounding rectangles, which were expanded by 15 pixels on each side to prevent truncation. Images were then split into 12 single-lead channels and stored in TIFF format to optimize batch reading efficiency. For model input, images were rescaled to 512 × 512 pixels, and grayscale values were linearly normalized to [0,1].

### 2.3 Data partitioning and augmentation

Each patient contributed a single index 12-lead ECG to the dataset; therefore, each ECG image corresponded to a unique patient. The dataset was partitioned at the patient level into training and testing subsets at a 7:3 ratio (Table 1), ensuring that data from the same patient did not appear in both subsets. Baseline demographic variables, including age and sex, were statistically analyzed using IBM SPSS Statistics for Windows, Version 27.0 (IBM Corp., Armonk, NY, United States). No significant differences were observed between the two subsets (*p* > 0.05), indicating that the allocation process maintained demographic balance.

**Table 1:** Data distribution of training set and test set.

**Type**	**Feature**	**Training set**	**Test set**	***p*-Value**
Normal	Male	96/186 (51.6%)	41/79 (51.9%)	0.966
	Average age (years)	46.8 ± 16.5	45.1 ± 14.7	0.422
STEMI	Male	84/109 (77.1%)	36/46 (78.3%)	0.871
	Average age (years)	64.1 ± 13.9	63.4 ± 13.5	0.778
NSTEMI	Male	159/202 (78.7%)	69/86 (80.2%)	0.771
	Average age (years)	63.1 ± 12.0	64.2 ± 11.2	0.476
UA	Male	88/144 (61.1%)	35/61 (57.4%)	0.618
	Average age (years)	65.3 ± 12.5	66.0 ± 12.0	0.744
AD	Male	149/193 (77.2%)	64/82 (78%)	0.878
	Average age (years)	56.2 ± 12.6	57.4 ± 12.6	0.464

To improve model generalizability and robustness, data augmentation was performed by applying horizontal translations of 1, 2, 3, 5, and 10 pixels to the ECG images during the training phase, simulating minor positional variations that may occur during signal acquisition. Importantly, augmentation was applied only after the train-test split and only to the training subset, resulting in a fivefold increase in the effective training dataset. The test subset contained only the original (non-augmented) ECGs and underwent only normalization to ensure an unbiased evaluation.

### 2.4 Deep learning model design and evaluation

**Figure 1:**
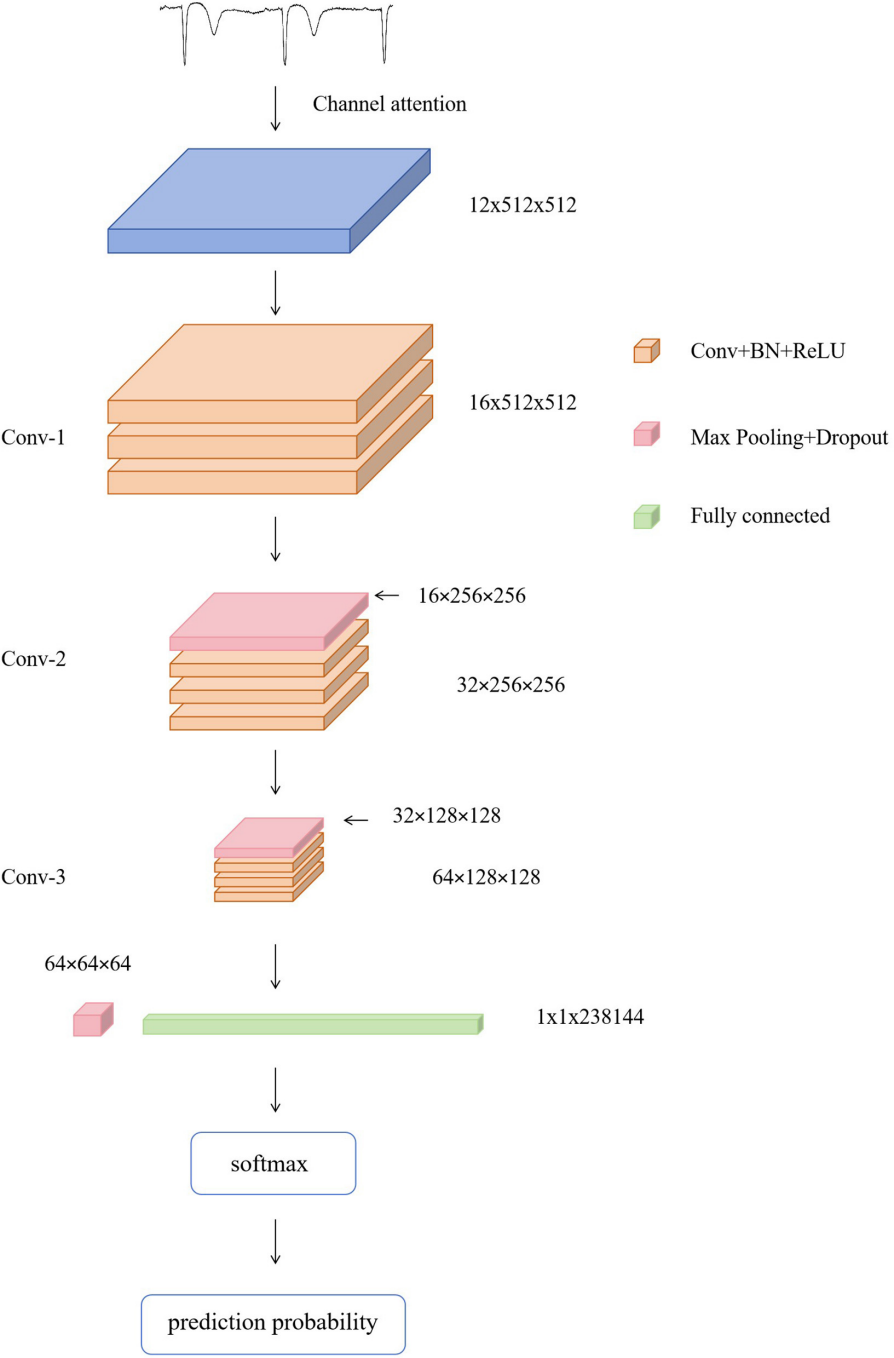
Model structure.

The proposed deep learning framework was implemented in Python using the PyTorch library (Figure 1). A convolutional neural network architecture was designed to process ECG tensors with dimensions of 12 × 512 × 512, corresponding to 12 leads and spatially normalized image inputs. The network consisted of a channel attention module and three convolutional blocks:

Channel attention was implemented as an SE-like module to recalibrate feature channels. Given a feature map *x*∈ℝ^*n*×12 × 512 × 512^, global average pooling was applied to obtain a channel descriptor *z*∈ℝ^*n*×12^. Two fully connected layers with a reduction ratio *r* and a ReLU non-linearity were then used to generate channel-wise weights *s*∈ℝ^*n*×12^ through a sigmoid gate, i.e., *s* = σ(*W*_2_δ(*W*_1_*z*)). The weights were reshaped to ℝ^*n*×12 × 1 × 1^ and applied by channel-wise multiplication with broadcasting, yielding the recalibrated feature map *x*′ = *x*⊙*s*.

The attention output was followed by three convolutional blocks. Each block used Conv2D–BatchNorm–ReLU with kernel size 3 × 3, convolution stride 1, and padding 1 (“same”). After each block, MaxPooling was applied with kernel size 4 × 4 and stride 2, and Dropout2D with *p* = 0.1 for regularization. The three convolution layers used channel dimensions 12 → 16, 16 → 32, and 32 → 64, respectively.

Each convolutional block followed a Conv2D–BatchNorm–ReLU design. All convolution layers used a stride of 1, with “same” padding to preserve spatial resolution before pooling. Max-pooling (2 × 2) was applied after convolutional blocks to progressively downsample feature maps, and dropout was used in the intermediate stage to reduce overfitting. Flattened feature maps were passed through fully connected layers and classified using a Softmax output layer.

The model was trained with an Adam optimizer (initial learning rate = 1 × 10^−4^, weight decay = 1 × 10^−4^), batch size of 8, for up to 100 epochs, with CrossEntropyLoss. A dynamic checkpointing strategy based on validation AUC was applied. Performance was assessed using accuracy, precision, recall, F1-score, AUC, average precision (AP), and confusion matrices. Inference time per case was also recorded.

### 2.5 Comparison with physician interpretation

Two experienced physicians independently performed blind ECG interpretation on the test set in a standard reading environment. To minimize memory effects and prior bias, a single set of ECGs was sampled from the overall cohort with approximately similar numbers across categories, resulting in 550 ECGs for the physician experiment. During manual interpretation, quantitative electrophysiological indices such as ST-segment deviation and Q-wave duration were systematically documented for diagnostic reference.

Confusion matrices, accuracy, precision, and recall were calculated for each physician and compared with AI results. Interpretation time per case was recorded, and statistical comparisons were performed using appropriate methods, with *post-hoc* analyses conducted using the Games–Howell test.

## 3 Results

### 3.1 Model performance and confusion matrices

The confusion matrices for each disease category (Tables 2–5) reveal that the deep learning model achieved excellent performance in recognizing STEMI, with minimal misclassification and both high sensitivity and specificity. For NSTEMI, the model maintained strong discriminatory ability. In contrast, performance for UA was comparatively suboptimal. Although the model successfully detected the majority of UA cases, it generated a considerable number of false positives, resulting in high recall but relatively low precision. Similarly, for AD, the model achieved only moderate accuracy, correctly identifying many true cases but producing frequent misclassifications, particularly between AD and normal ECG patterns. Collectively, these results indicate that the proposed model is highly reliable for STEMI and reasonably effective for NSTEMI, but its performance is limited for UA and AD. The comparatively lower performance for UA and AD is consistent with clinical practice, where these conditions typically require biomarker or imaging confirmation. Therefore, the ECG-only results should be interpreted as the achievable discrimination level from ECG-report images alone.

**Table 2:** Confusion matrix for STEMI (*n* = 125).

**Actual/Predicted**	**STEMI**	**Normal**	**Total**
STEMI	42	4	46
Normal	1	78	79
Total	43	82	125

**Table 3:** Confusion matrix for NSTEMI (*n* = 165).

**Actual/Predicted**	**NSTEMI**	**Normal**	**Total**
NSTEMI	77	9	86
Normal	9	70	79
Total	86	79	165

**Table 4:** Confusion matrix for UA (*n* = 140).

**Actual/Predicted**	**UA**	**Normal**	**Total**
UA	52	9	61
Normal	35	44	79
Total	87	53	140

**Table 5:** Confusion matrix for AD (*n* = 161).

**Actual/Predicted**	**AD**	**Normal**	**Total**
AD	55	27	82
Normal	32	47	79
Total	87	74	161

As summarized in Table 6, the proposed deep learning model achieved the highest diagnostic performance in the STEMI category. For STEMI, the model achieved an accuracy of 96.0%, with precision, recall, F1-score, and AUC of 0.977, 0.913, 0.944, and 0.986, respectively. In the NSTEMI category, the model attained an accuracy of 89.1% and an AUC of 0.916, further confirming its reliable generalization and stable classification performance.

**Table 6:** Summary of performance metrics across disease categories.

**Category**	**Accuracy (%)**	**Precision**	**Recall**	**F1-score**	**AUC**	**AP**
STEMI	96.0	0.977	0.913	0.944	0.986	0.987
NSTEMI	89.1	0.895	0.895	0.895	0.916	0.945
UA	68.6	0.598	0.853	0.703	0.827	0.450
AD	63.4	0.632	0.671	0.651	0.756	0.691

In contrast, the performance for UA and AD was less satisfactory. Although the model retained a relatively high recall for UA (0.853), its precision dropped markedly to 0.598, reflecting substantial false positives. Similarly, the classification of AD yielded only moderate precision (0.632) and recall (0.671), accompanied by a lower AUC of 0.756, reflecting the difficulty in distinguishing AD from normal or ischemic ECG patterns.

These results collectively suggest that the model is highly accurate and clinically applicable for detecting STEMI and NSTEMI, but its effectiveness in recognizing UA and AD remains limited due to overlapping or non-specific ECG features.

### 3.2 Comparison with physician interpretation

As presented in Table 7, the average interpretation time per case differed markedly between the AI model and physicians. The average inference time of AI for 591 ECGs was 0.24 ± 0.08 s, which was significantly faster than Physician A (23.84 ± 9.45 s, *n* = 550) and Physician B (22.98 ± 9.06 s, *n* = 550; *p* < 0.001). Statistical analysis using the Games–Howell *post-hoc* test confirmed significant differences between the AI model and both physicians (*p* < 0.001), whereas no significant difference was observed between the two physicians (*p* = 0.278). It is worth noting that the same normal ECGs from the test split were reused across multiple disease-vs.-normal evaluations. Therefore, the test sample size exceeds the unique test count shown in the split summary.

**Table 7:** Comparison of average interpretation time.

**Group**	***N***	**Reading time (s)**	***p*-Value**	**Games–Howell (** ***p*** **)**		
				**AI**	**Physician A**	**Physician B**
AI	591	0.24 ± 0.08	< 0.001	—	< 0.001	< 0.001
Physician A	550	23.84 ± 9.45		< 0.001	—	0.278
Physician B	550	22.98 ± 9.06		< 0.001	0.278	—

As shown in Table 8, the diagnostic performance of the AI model was comparable to or exceeded that of physicians in identifying STEMI and NSTEMI. For STEMI, the AI system achieved an accuracy of 96.0% with the high precision (0.977), while maintaining recall levels similar to those of the physicians, indicating strong diagnostic consistency. In the detection of NSTEMI, AI performance was generally balanced, indicating reliable generalization.

**Table 8:** Performance comparison between AI and physicians.

**Category**	**Metric**	**AI**	**Physician A**	**Physician B**
STEMI	Accuracy (%)	96.0	94.5	93.5
	Precision	0.977	0.936	0.976
	Recall	0.913	0.957	0.891
NSTEMI	Accuracy (%)	89.1	93.6	91.3
	Precision	0.895	0.941	0.918
	Recall	0.895	0.930	0.907
UA	Accuracy (%)	68.6	79.5	83.6
	Precision	0.598	0.810	0.860
	Recall	0.853	0.770	0.803
AD	Accuracy (%)	63.4	65.2	67.1
	Precision	0.632	0.821	0.804
	Recall	0.671	0.390	0.451

In contrast, physician interpretation outperformed AI in the recognition of UA and AD. Although the AI model yielded higher recall for UA (0.853), its precision was markedly lower (0.598), reflecting a high rate of false positives. Similarly, for AD, the AI achieved moderate precision (0.632) and recall (0.671), whereas both physicians demonstrated better overall accuracy and precision despite somewhat lower recall.

Collectively, these findings suggest that the AI system can match or even surpass human expertise in rapidly identifying STEMI and NSTEMI, but remains inferior to experienced physicians in the nuanced classification of UA and AD, where non-specific or overlapping ECG features present greater interpretive challenges.

## 4 Discussion

In recent years, deep learning has demonstrated remarkable progress in medical image analysis, particularly in large-scale ECG interpretation tasks, where it shows strong feature extraction and classification capabilities (13, 14). Previous studies have successfully constructed deep learning models for ECG analysis that could localize culprit vessels in STEMI with near-perfect accuracy (AUC up to 0.998), underscoring the potential of AI to achieve cardiologist-level diagnostic performance (15). These findings collectively highlight that deep learning algorithms are well-suited for processing complex physiological signals and can play a pivotal role in clinical decision-support systems aimed at enhancing diagnostic speed and consistency (16, 17). In this study, the proposed CNN-based model achieved rapid and accurate recognition of STEMI and NSTEMI, demonstrating significant clinical potential for triage and early decision-making in patients with acute chest pain. Notably, the average inference time was less than 1 s per case, substantially faster than human interpretation, thereby emphasizing the model's potential for real time deployment in emergency settings where time-sensitive decision-making is crucial.

However, the model's performance in UA and AD classification was comparatively suboptimal. This limitation can be attributed to the inherent clinical and electrophysiological characteristics of these conditions. UA often exhibits transient or subtle ECG abnormalities, such as intermittent ST-segment depression or T-wave abnormalities, and may appear normal during asymptomatic periods. Similarly, AD is a structural aortic disorder whose ECG findings are typically non-specific, reflecting secondary ischemia or conduction disturbances rather than disease-specific patterns (18). Consequently, the overlap between these ECG patterns and those of normal individuals reduces the model's precision, leading to more false positives despite high recall. This observation highlights the intrinsic challenge of single-modality ECG analysis in detecting diseases with ambiguous or indirect electrophysiological signatures.

ECG interpretation in emergency settings is challenging because waveform morphology can be influenced by confounding patterns, and patients may present with overlapping or mixed etiologies that blur diagnostic boundaries when relying on ECG alone. In this study, the final confirmed clinical diagnosis was used in the electronic medical record after routine work-up as the reference standard. To ensure a single label per case, each patient contributed one index ECG and the documented primary diagnosis was used as the reference label. Cases with ambiguous diagnoses or unreadable ECG reports were excluded by predefined criteria. This indicates that an ECG-only setting cannot fully resolve mixed etiologies or eliminate non-specific ECG effects. Accordingly, this study quantified the achievable discrimination level from ECG-report images under real-world data constraints, as an initial benchmark for triage-oriented decision support.

Considering these limitations, future work should emphasize multimodal data integration. In clinical research, multimodal AI approaches have shown substantial promise in improving diagnostic accuracy and disease subtyping. For example, combining multiple imaging modalities and clinical data has enhanced diagnostic precision in urological diseases (19), while multimodal ensemble deep learning frameworks integrating CT and PET have successfully predicted HPV status in head and neck cancers (20). By analogy, integrating 12-lead ECG data with cardiac biomarkers (e.g., high-sensitivity troponin), echocardiographic findings, and computed tomography angiography could further refine diagnostic specificity and reduce false positives. Such integration may lead to more comprehensive and reliable classification models, ultimately improving diagnostic accuracy and clinical decision-making for acute chest pain in emergency department. In addition, the model was trained on image-form ECG reports rather than raw numerical waveforms, which may contain richer temporal precision and metadata. Future work should evaluate raw-signal models when waveform files are consistently accessible. Moreover, as a single-center study, the evaluation constitutes internal validation. External and temporal validation should be performed to confirm generalizability across institutions, devices, and time periods when additional datasets become available.

## 5 Conclusions

This study developed a deep learning model based on 12-lead ECGs for the rapid classification of acute chest pain. The model achieved excellent performance in detecting STEMI and NSTEMI with high accuracy and near-instantaneous interpretation, supporting the feasibility and utility of AI-assisted ECG interpretation in emergency chest pain management. In contrast, recognition of UA and AD was limited, underscoring the constraints of ECG as a single modality. Future work should integrate multimodal clinical data and multi-center cohorts to enhance generalizability. Overall, AI-assisted ECG interpretation shows strong promise as an efficient decision-support tool in emergency department.

## Data Availability

The original contributions presented in the study are included in the article/supplementary material, further inquiries can be directed to the corresponding author.
